# Impact of the COVID-19 Pandemic on Patient Preferences and Decision Making for Symptomatic Urolithiasis

**DOI:** 10.1089/end.2020.1141

**Published:** 2021-08-12

**Authors:** Tommy Jiang, Vadim Osadchiy, James M. Weinberger, Michael H. Zheng, Michael H. Owen, Sarah A. Leonard, Jesse N. Mills, Naveen Kachroo, Sriram V. Eleswarapu

**Affiliations:** ^1^ Division of Andrology, Department of Urology, David Geffen School of Medicine, University of California, Los Angeles, Los Angeles, California, USA.; ^2^ Consortium for Health Activity on Social Media, David Geffen School of Medicine, University of California, Los Angeles, Los Angeles, California, USA.; ^3^ Department of Statistics, University of California, Berkeley, California, USA.; ^4^ Department of Computer Science, College of Liberal Arts, University of Iowa, Iowa City, Iowa, USA.; ^5^ College of Letters and Sciences, Carleton College, Northfield, Minnesota, USA.; ^6^ Glickman Urological and Kidney Institute, Cleveland Clinic, Cleveland, Ohio, USA.; ^7^ Department of Urology, Vattikuti Urology Institute, Henry Ford Hospital, Detroit, Michigan, USA.

**Keywords:** urolithiasis, natural language processing, COVID-19, patient perspectives

## Abstract

***Background:*** Pandemic restrictions have changed how patients approach symptomatic kidney stones. We used a mixed-methods digital ethnographic approach to evaluate social media discussions about patient concerns and preferences for urolithiasis care during the COVID-19 pandemic.

***Materials and Methods:*** We retrospectively analyzed kidney stone-related discussions on a large social media platform using qualitative analysis and natural language processing-based sentiment analysis. Posts were mined for demographic details, treatments pursued, and health care encounters. Pre-COVID-19 (January 1, 2020–February 29, 2020) and COVID-19 (March 1, 2020–June 1, 2020) posts were extracted from the popular online Reddit discussion board, “r/KidneyStones,” which is dedicated to discussions related to urolithiasis.

***Results:*** We extracted *n* = 649 posts (250 pre-COVID-19, 399 COVID-19); 150 from each cohort underwent thematic analysis and data extraction. Quantitative sentiment analysis was performed on 418 posts (179 pre-COVID-19, 239 COVID-19) that described stone-related decision making before intervention. Notable discussion themes during COVID-19 focused on barriers to care and concerns about stone management. Discussants exhibited more negative and anxious tones during COVID-19, based on sentiment analysis (*p* < 0.01). Patient preferences shifted away from in-person visits and procedures (*p* < 0.001). Mean reported stone size among those visiting emergency room (ER) increased from 5.1 to 10.5 mm (*p* < 0.001). The proportion of discussants preferring conservative management with stones ≥10 mm increased (12.5% pre-COVID-19 *vs* 26% during COVID-19, *p* = 0.002). Opioid mentions increased from 9% to 27% of posts (*p* < 0.001) and were most associated with conservative management discussions.

***Conclusions:*** Online discussion forums provide contemporaneous insight into patients' experiences during a time when traditional patient-centered research methodologies are limited due to social distancing. During the pandemic, patients with symptomatic kidney stones expressed anxiety regarding outpatient encounters and reluctance toward procedural intervention. Patients opted instead for at-home conservative treatment beyond clinical guidelines and reserved ER visits for larger stones, potentially causing self-harm. Opioid discussions proliferated, an alarming consequence of the pandemic.

## Introduction

The downstream impact of the COVID-19 pandemic on health care utilization remains an open question.^1^ At the outset of the pandemic, policies were adopted to limit infectious transmission, including stay-at-home orders, social distancing, and postponement of elective surgery.^2^ Such policies have contributed to a decrease in emergency room (ER) volumes,^3^ increased anxiety about COVID-19,^4,5^ and a rise in telehealth.^6^ Hospital systems have had to prioritize emergency surgeries and triage elective procedures.

It is unclear how the pandemic may have disrupted treatment pathways for kidney stone disease, which affects nearly 1 in 11 individuals and contributes to 1 million ER visits each year in the United States.^7,8^ Treatment options range from observation or medical expulsive therapy (MET) to operative interventions such as extracorporeal shock wave lithotripsy (SWL), ureteroscopy (URS), and percutaneous nephrolithotomy (PCNL).^9^ Patient preference, stone characteristics, and comorbidities weigh heavily in decision making.

Patient perspectives on how COVID-19 has changed health care utilization and decision making remain elusive. Social media platforms offer unique opportunities to understand patients' anxieties. Reddit (www.reddit.com) is one such forum, with 330 million monthly active users.^10^ Previous studies have leveraged these online discussions to evaluate topics such as infertility,^11^ erectile dysfunction,^12^ and suicide.^13^ The Reddit forum “r/KidneyStones” (www.reddit.com/r/KidneyStones/) contains >4000 active members and is devoted to urolithiasis.

We sought to understand how the COVID-19 pandemic has affected patients' decision making regarding kidney stones. Our patient-centered approach combined qualitative thematic analysis with quantitative natural language processing (NLP) of the r/KidneyStones discussion forum. We hypothesized that COVID-19 would cause discussants to favor options that minimized in-person hospital encounters, resulting in observation and MET for cases who traditionally undergo procedural intervention.

## Methods

### Data collection

To evaluate discussions on the Reddit r/KidneyStones community, we employed a mixed-methods approach involving (1) classic qualitative thematic analysis, (2) an NLP-based technique called Linguistic Inquiry and Word Count (LIWC), and (3) manual mining of a subset of discussions. Reddit was chosen because it contained a dedicated discussion board to urolithiasis, and was publicly available, anonymous, and had no limitations to content or word count.

We define a “post” as a discussant's initial textual entry. We retrospectively analyzed posts from r/KidneyStones from January 1, 2020 through June 1, 2020. We used February 29, 2020 as our cutoff between “Pre-COVID-19” and “COVID-19” periods since this corresponds to the first emergency declaration in the state of Washington.^14^ No posts in the pre-COVID-19 cohort discussed the pandemic.

### Qualitative thematic analysis

We performed qualitative thematic analysis of 150 randomly selected posts from the pre-COVID-19 and COVID-19 periods.^15^ This number was selected to achieve thematic saturation, whereby no additional themes were identified. We used grounded theory and constant comparative methodology.^16,17^ Two investigators independently analyzed each post to identify preliminary themes, which were then finalized among the study authors.^15^

### NLP-derived sentiment analysis

We utilized LIWC2015, a previously validated^11,18^ application that analyzes textual data based on quantitative metrics related to psycholinguistic parameters.^19^ For sentiment analysis, we used four summary variables (analytical thinking, clout, authenticity, and emotion) and three emotional tone-based variables (anxiety, anger, and sadness) to evaluate decision making. These variables were validated from data sets comprising large comparison samples.^19,20^ Scores for these variables range from 0 to 100 (50 indicates a neutral tone). LIWC's validated dictionary includes an index that categorizes words as having specific valences. Higher scores indicate the presence of a larger proportion of LIWC's indexed words that express those valences in a post.

Higher analytical thinking suggests language that is more formal/logical. Higher clout suggests that the writer speaks from the perspective of an expert. Higher authenticity reflects a more honest/straightforward style. For emotion, higher scores reflect more positive tones. Sentiment analysis was performed solely on posts that described stone-related decision making before intervention, since this group of individuals were likely to be strongly affected by the pandemic.

### Textual data extraction and mining

We extracted the following data: demographics (age and gender), relevant medical history (stone size, pre- or poststone passage status, and history of urolithiasis), health care visits (ER, primary care physician, or urologist), treatment modalities discussed (observation, MET, SWL, URS, and PCNL), and discussion of opioids or nonopioid medication. Descriptive statistics were calculated, and comparisons were made between the pre-COVID-19 and COVID-19 periods.

### Statistical analysis

For NLP-based LIWC sentiment analysis, Mann–Whitney *U* tests compared means of variables between pre-COVID-19 and COVID-19 posts. For analysis of manually extracted data, two-sided two-sample proportions tests were used for categorical variables, and two-sided Student *t*-tests were used for continuous variables. Univariate analysis was conducted for stone size in relation to provider type, pain medication, and treatment modality. RStudio version 1.1.463 (RStudio, Inc., Boston, MA) was used for statistical analyses, with *p* < 0.05 considered statistically significant.

## Results

We extracted 649 posts: 250 pre-COVID-19 and 399 COVID-19. Of these, 179 pre-COVID-19 and 239 COVID-19 posts underwent NLP analysis. A random selection of 150 posts from each period (300 total) underwent qualitative thematic analysis and manual data extraction and mining.

### Qualitative thematic analysis

Themes were divided into pre-COVID-19, COVID-19, and themes common to both.

#### (Pre-COVID-19) desiring second opinion

Social media served as a resource to help patients decide on a treatment option and get clarification about their conditions after visits to health care providers.

“Scheduled ureteroscopy in an effort to pass them without surgery […] but the urologist seemed concerned that I wouldnt be able to pass the stones and may be in pain until the 6^th^ […] I'm curious to see if any of you all had similar experiences or words of wisdom.”

“I'm not a doc so I have no idea what the difference is between these two tests, why they showed different things, or which is more accurate…. What do y'all think?”

#### (Pre-COVID-19 and COVID-19) general education about kidney stones

Discussants turned to this forum to learn more about urolithiasis.

“There was a lot of pain in my left kidney area. They confirmed blood in my urine. I was vomiting… Now it is Tuesday and I all of a sudden feel a stinging pain down by my urethra opening. Could this be the stone?”

Discussants sought advice to manage expectations.

“I don't know what to expect from this procedure (ESWL) as I've never had any invasive procedures done for removing stones […] how long does recovery take so I can resume my daily activities?”

#### (Pre-COVID-19 and COVID-19) reflections after stone passage or procedural intervention

Individuals wrote extensively about their experiences with urolithiasis, often reflecting on side effects of treatment.

“So lithotropy sucks…. Had two stones blasted today via a ureteroscopy. Had my first pee at the hospital and literally thought I was going to cry from the burning sensation coming from my equipment […] I almost prefer the back and flank pain right now.”

#### (COVID-19) concerns and anxiety about kidney stone management

Discussions taking place during COVID-19 highlighted concerns with urolithiasis management. Uncertainty was often related to the pandemic.

“Now I know most would have prefer the surgery but I'm also afraid of going to the hospital. Our county currently has 39 positive cases. 8 in my city. I know that's not many but on Thursday we had 19.”

Numerous individuals changed treatment plans to home-based management. Discussions centered on anecdotal experiences with home-based remedies.

“Due to COVID I obviously haven't been able to be with him during this one […] I don't know if this is normal or not […] I'm worried I might not make the right decisions in helping and caring for him once he gets back home.”

#### (COVID-19) barriers in accessing kidney stone care

Discussions during COVID-19 focused on barriers in accessing kidney stone care. Individuals felt unsafe when visiting a health care provider for evaluation.

“First attack was awful. This final attack was just so bad I can't see even a strong narcotic being able to help […] Can anyone attest to hard drugs like this actually working so I won't have to go to the ER at a time like this?”

Individuals had treatment plans altered, with elective procedures being cancelled and replaced with MET. Others sought advice on navigating the health care system during the pandemic.

“26f with a 10mm stone […] Should I go right to the ER or wait a few days to see if this means I'm going to pass it? All they gave me was flomax and oxycodone.”

“I'm having trouble finding a urologist nearby due to some offices being closed due to COVID but one that did pick-up said they would need a referral […] I've tried calling walk in clinics but none of the them were equipped with A CT machine or ultrasound.”

Patients also faced barriers when they were in the hospital. Decisions had to be made without input from family due to hospital policies and social distancing protocols.

“I went to the ER two days ago, with piercing pain in the lower right part of my back […] So I had a few basic questions that I couldn't get answered at the ER, because with Coronavirus I couldn't have a family member to help me ask questions.”

### Quantitative thematic analysis

Results of LIWC are given in Table 1. Discussants during COVID-19 were more likely to exhibit a negative, anxious, and authentic tone, and less likely to write confidently.

**Table 1.** Language Inquiry and Word Count Results for Preintervention Kidney Stone Social Media Posts

Valence	Z-score	p
Anxiety	3.34	<0.01
Anger	1.78	NS
Sadness	0.99	NS
Analytical	−0.95	NS
Clout	−3.02	<0.01
Authenticity	2.46	<0.05
Tone	−2.83	<0.01

Social media discussions about kidney stones before and during the COVID-19 pandemic underwent sentiment analysis. Four summary valences (analytical thinking, clout, authenticity, and emotion) and three emotional valences (anxiety, anger, and sadness) were selected for this analysis. *Z*-scores represent the degree to which pre-COVID-19 posts and COVID-19 posts differ for a specified valence. A negative *Z*-score indicates that the valence was more prevalent in pre-COVID-19 discussions, whereas a positive *Z*-score signifies language more prevalent in the COVID-19 period. Mann–Whitney *U* Tests were used to calculate *p*-value, with *p* < 0.05 considered statistically significant.

NS = not significant.

### Manual data extraction and mining

#### Patient characteristics

Demographic data are given in Table 2. No difference was noted in user-reported age or gender between pre-COVID-19 and COVID-19. No difference was observed in stone size, stone passage status, or history of urolithiasis.

**Table 2.** Characteristics of Discussants, Medical History, and Kidney Stone Episodes Described in Social Media Posts Pre-COVID-19 and During COVID-19

	Pre-COVID-19		COVID-19		
	Total (*n* = 150)	%	Total (*n* = 150)	%	p
Author					
Patient	149	99	145	97	NS
Other	1	1	5	3	NS
Age (years)	*n = 34*		*n = 45*		
Mean (SD)	31.0 (13.5)		29.4 (8.7)		NS
Median (IQR)	25 (21–40.5)		27 (24–33)		
Gender					
Male	27	18	28	19	NS
Female	20	13	22	15	NS
Unknown	103	69	100	67	NS
Temporal association of post					
Prestone passage	116	77	108	72	NS
Poststone passage	34	23	42	28	NS
Stone episode					
First episode	40	27	39	26	NS
Recurrent episode	48	32	42	28	NS
Unknown	62	41	69	46	NS
Stone size (mm)	*n = 66*		*n = 77*		
Mean (SD)	7.5 (5.8)		7.1 (3.9)		NS
Median (IQR)	6 (4–9)		6.5 (4–9)		

Demographic data (author, age, and gender) and relevant medical history (temporal association of post, stone episode, and stone size) were extracted to compare social media posts pre-COVID-19 and during COVID-19. Two-sided two-sample proportions test was used to calculate significance, with *p* < 0.05 considered statistically significant.

IQR = interquartile range; SD = standard deviation.

#### Discussions about health care provider visits

**FIG. 1.**
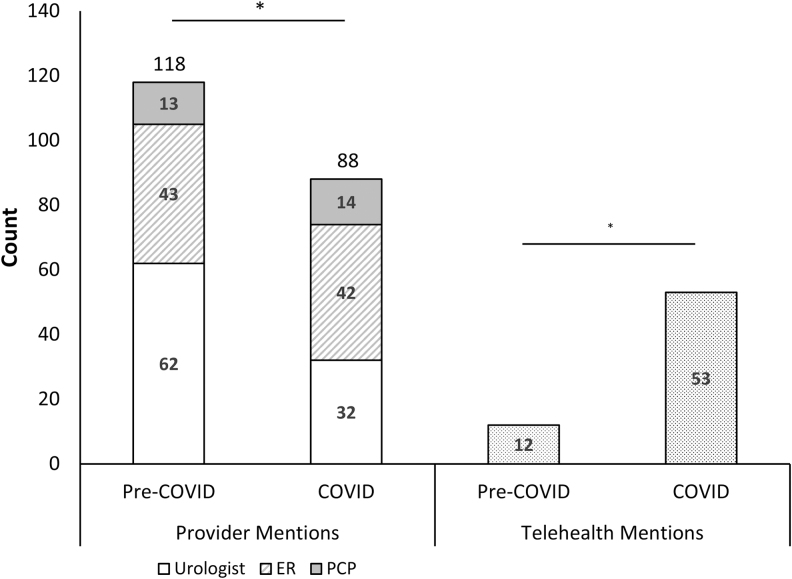
Self-reported provider preferences of social media discussants. Discussants on social media expressed preferences for the types of health care encounters they were comfortable with pursuing for kidney stone evaluation and management. Provider mentions and the use of telehealth were quantified during data mining. Total provider mentions were then further categorized into urologist, ER, and PCP. Provider type was double counted if multiple providers were mentioned in a post. Two-sided two-sample proportions test was used to determine whether there was a difference between the pre-COVID-19 period and the COVID-19 period, with *p* < 0.05 considered statistically significant. *Denotes statistically significant differences. Overall total provider mentions declined in social media posts between the two periods. The proportion of urologist mentions decreased and proportion of ER mentions increased. ER = emergency room; PCP = primary care physician.

Forum users discussed visiting a health care provider at a significantly higher rate during the pre-COVID-19 period (118/150, 78.6%) compared with during COVID-19 (88/150, 58.7%), *p* < 0.001. Within these discussions of health care providers, the proportion of ER mentions increased from 36% (43/118) to 48% (42/88) (*p* < 0.001), whereas urologist mentions decreased from 53% (62/118) to 36% (32/88), *p* < 0.001 (Fig. 1).

In 44 posts—24 pre-COVID-19 and 20 COVID-19—discussants provided data on stone size and described visiting the ER for pain. Stone sizes associated with ER visits were 5.1 ± 2.8 mm (pre-COVID-19) and 10.5 ± 4.4 mm (COVID-19), *p* < 0.001 (Table 3).

**Table 3.** Relationship of Kidney Stone Size with Health Care Provider Encounters Mentioned on Social Media

	Pre-COVID-19		COVID-19		
	N	Stone size (mm)	N	Stone size (mm)	p
Possibility of seeking medical attention is discussed	56	7.6 (5.3)	61	7.9 (4.0)	NS
Urologist	39	8.8 (5.7)	23	8.1 (4.7)	NS
ER	**25**	**5.1 (2.7)**	**36**	**9.4 (4.3)**	**<0.01**
Primary care	7	4.1 (1.2)	8	7.8 (6.4)	NS
Health care encounter was described to have actually occurred	56	7.6 (5.3)	43	8.5 (4.3)	NS
Urologist	37	9.0 (5.8)	19	8.6 (4.9)	NS
ER	**24**	**5.1 (2.8)**	**20**	**10.5 (4.4)**	**<0.01**
Primary care	7	4.14 (1.2)	7	7.1 (6.6)	NS

Bold values indicate significant differences.

We evaluated social media posts by discussants who reported kidney stone sizes in the pre-COVID-19 period and the COVID-19 period to determine whether the pandemic affected decision making about seeking medical attention. Providers could be double counted if multiple visits or mentions were stated in a post. The stone size warranting ER evaluation increased during the COVID-19 period. Univariate analysis using two-sided Student *t*-tests was employed, with *p* < 0.05 considered statistically significant.

ER = emergency room.

#### Self-reported treatment preferences

**FIG. 2.**
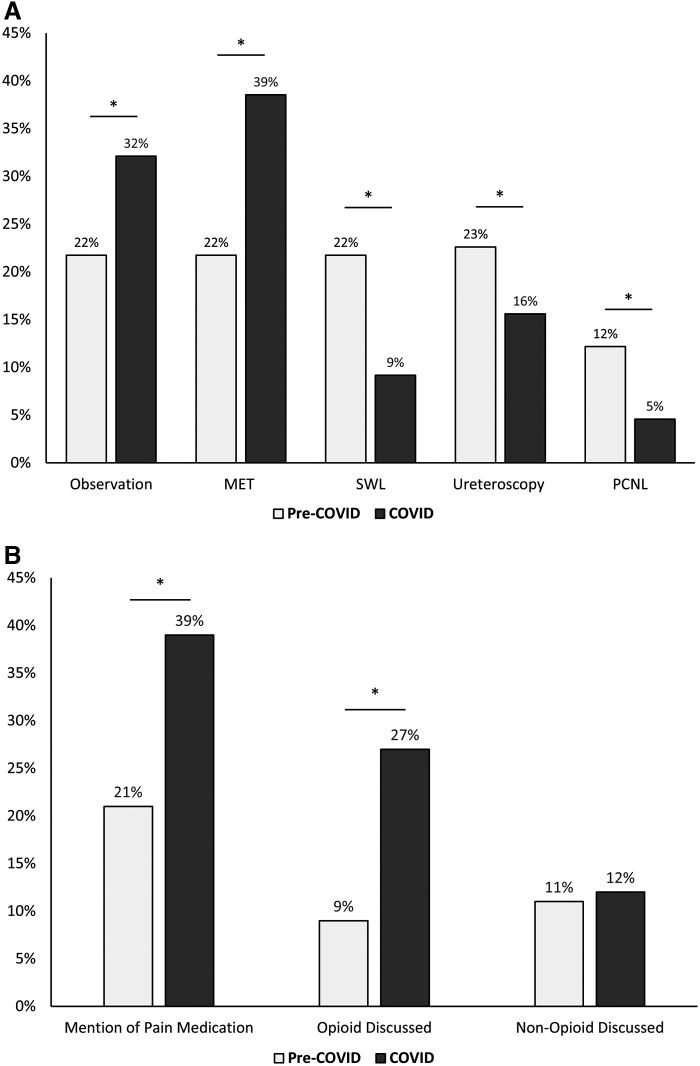
Self-reported kidney stone treatment preferences among social media discussants. **(A)** Mentions of treatment modalities (observation, MET, SWL, ureteroscopy, or PCNL) were quantified during textual data extraction from social media posts. In total, treatments were mentioned 115 and 109 times during the pre-COVID-19 and COVID-19 periods, respectively. Two-sided two-sample proportions test was used to assess differences between the periods. Discussions of observation and MET increased during COVID-19, whereas discussions of procedural interventions decreased. **(B)** Likewise, mentions of oral analgesic control (stratified into opioid or nonopioid) were quantified in the pre-COVID-19 and COVID-19 periods. Two-sided two-sample proportions test was used to assess a difference between the periods. During COVID-19, overall discussions of analgesia nearly doubled, and the proportion of discussions about opioid medications tripled. For all tests, *p* < 0.05 constituted statistical significance. *Denotes statistically significant differences. MET = medical expulsive therapy; PCNL = percutaneous nephrolithotomy; SWL = extracorporeal shock wave lithotripsy.

Among forum discussants, self-reported preferences included observation, MET, SWL, URS, or PCNL. Observation and MET significantly increased from the pre-COVID-19 period to the COVID-19 period, whereas operating room interventions (SWL, URS, and PCNL) significantly decreased (Fig. 2A). Among discussants who preferred observation or MET and reported a stone size, the proportion with stone sizes ≥10 mm increased from 12.5% (4/32) pre-COVID-19 to 26% (13/50) in the COVID-19 period, *p* = 0.002.

From pre-COVID-19 to COVID-19, there was a threefold increase in opioid mentions among discussion posts (*p* < 0.001) (Fig. 2B). This increase was most prominent in discussants undergoing observation or MET (25% pre-COVID-19 to 60% during COVID-19, *p* < 0.001).

## Discussion

Health system changes during the COVID-19 pandemic have shifted patient approaches to disease. Traditional patient-centered research methodologies are limited during pandemic social distancing. To obtain contemporaneous insights into patients' experiences in a rapidly changing environment, we evaluated posts on a social media platform to identify drivers of decision making regarding kidney stones. Our results suggest that kidney stone treatment preferences during COVID-19 are driven by barriers to surgical access and anxieties about infection. Two notable findings during COVID-19 were that opioid-related discussions dramatically increased, and MET proliferated beyond its guideline-based indication.

Opioid mentions tripled during the COVID-19 period, suggesting a shift toward at-home symptom control rather than hospital-based definitive management. Typically, nonsteroidals are first-line analgesics for kidney stones 5–10 mm,^21^ followed by opioids. The American Urological Association Guideline advises urology consultation for procedural intervention for ureteral stones ≥10 mm to prevent upper urinary tract damage that may occur otherwise.^22^ In contrast, we found that the percentage of discussants preferring noninvasive management for stones ≥10 mm more than doubled, and there was an associated increase in opioid interest for these larger stones. Taken together with the higher anxiety uncovered in semantic analysis of language used during COVID-19, these data may reflect patient concerns regarding insufficient at-home analgesic control. Urolithiasis represents the top diagnosis associated with opioid prescribing among ER visits,^23^ despite opioids' debatable utility compared with nonsteroidals for stone pain.^24,25^ Further retrospective investigations will bear out whether the pandemic has led to increased opioid use among patients deferring procedural management for acute conditions. As of October 2020, 41 states and the District of Columbia have reported rises in opioid-related mortality during the pandemic.^26^

Thematic analysis highlighted barriers to care before, during, and after management of a discussant's kidney stone during the pandemic and, importantly, provided first-hand narratives of patients' perspectives. Barriers included safety concerns, lack of provider access, changes in hospital policies, and concerns with adequate treatment during COVID-19. Semantic analysis revealed higher levels of anxiety and uncertainty in the language used by discussants. Taken together, the qualitative and semantic analyses contribute patient-centered evidence regarding mental health in the pandemic, during which rates of self-reported depression and anxiety due to COVID-19 have been reported at 16%–28%.^27^ The mental health crisis emerging from the pandemic may prove to be considerable.^28^

During COVID-19, discussants were less likely to mention health care providers. There was a decline in the proportion of urologist mentions, consistent with outpatient data showing a 63% decrease in urology visits by April 2020 from prepandemic volumes.^29^ The proportion of ER mentions increased, and self-reported stone sizes for individuals presenting to the ER doubled during COVID-19. Our findings suggest that patients were overall less inclined to see a provider for kidney stones during the pandemic and only the most severely affected patients sought ER evaluation. Treatment preferences shifted toward medical management, and discussions of surgical interventions decreased. This is consistent with reported experiences among health care institutions, which saw declines in surgical volumes during the pandemic (e.g., a 71.7% decrease at Harborview Medical Center in Seattle, WA).^30^ These data highlight the power of social media as a tool for understanding patient decision-making trends.

Our study is not without limitations. Although social media platforms provide anonymity for honest discussions that shed light on decision making, key demographic data were unavailable. Data on discussants' geographic locations, stone position (e.g., collecting system or ureter), and other factors were not consistently available. Posts represent snapshots in time, limiting the scope of analysis to exclude future decision making and long-term outcomes. Finally, patients who use social media for as a decision aid may have categorically different concerns than patients seen in clinic.

## Conclusions

Our study highlights the effects of a pandemic on health care engagement, using social media to provide contemporaneous insights into patient experiences with kidney stones. The results underscore amplified patient anxiety and a pattern of reluctance among individuals who would have visited a physician for urolithiasis were it not for the pandemic. Patients are opting for at-home management of kidney stones that would previously have prompted ER evaluation. Opioid discussions among patients have increased. These trends contrast with society-based guidelines for kidney stone management. Physicians would do well to include online discussions within their armamentarium to gain additional nuance in patient decision making.

## Abbreviations Used

ER: emergency room
IQR: interquartile range
LIWC: language inquiry and word count
MET: medical expulsive therapy
NLP: natural language processing
PCNL: percutaneous nephrolithotomy
PCP: primary care physician
SD: standard deviation
SWL: extracorporeal shock wave lithotripsy
URS: ureteroscopy
